# Emergency Department to Inpatient Care Transition: A Narrative Review of Handover Failures, Diagnostic Momentum, and Patient Safety

**DOI:** 10.7759/cureus.109294

**Published:** 2026-05-20

**Authors:** Abhishek Hanumanpratap Singh Kshatri

**Affiliations:** 1 Emergency Medicine, Apollo Hospitals, Visakhapatnam, IND

**Keywords:** acute medical admissions, diagnostic errors, diagnostic momentum, emergency department, i-pass, medication reconciliation, patient handover, patient safety, sbar, transitions of care

## Abstract

The transition of care from the emergency department (ED) to inpatient services is a high-risk clinical boundary in acute medical admissions. During this process, the patient, clinical information, diagnostic reasoning, therapeutic responsibility, pending investigations, and escalation plans must move together. When this transfer is incomplete, patients may be vulnerable to missed results, medication discrepancies, unclear ownership, delayed escalation, and diagnostic momentum, where a provisional ED label becomes accepted by subsequent teams without adequate reassessment.

This narrative review examines handover failures, diagnostic momentum, and patient-safety risks during ED-to-inpatient care transitions. It also discusses how these transitions differ across health-system structures, including hospitalist-based models, acute medical take pathways, consultant-unit systems, ward transfers, high-dependency units (HDU), and intensive care settings. Common vulnerabilities include incomplete communication of illness severity, working diagnosis, diagnostic uncertainty, treatment response, pending tests, medication risks, and contingency plans. Structured communication tools such as SBAR (Situation, Background, Assessment, Recommendation) and I-PASS (Illness Severity, Patient Summary, Action List, Situation Awareness and Contingency Planning, Synthesis by Receiver) may improve reliability when adapted to local workflows, supported by training, and paired with receiver confirmation.

The review proposes practical handover elements for acute medical admissions, emphasizing illness severity, key differentials, pending tasks, medication reconciliation, escalation triggers, and explicit accountability for follow-up. The ED-to-inpatient transition should be treated as an active patient-safety intervention rather than a purely administrative movement of the patient. Clear communication of uncertainty, trajectory, and responsibility may reduce preventable harm during acute medical admissions.

## Introduction and background

The emergency department (ED) is one of the most important entry points for acutely ill medical patients. Patients with sepsis, acute coronary syndrome, heart failure, stroke-like symptoms, diabetic emergencies, acute kidney injury (AKI), electrolyte disturbances, gastrointestinal bleeding, and altered mental status often receive their first structured diagnostic and therapeutic pathway in the ED. Handoffs are recognized patient-safety vulnerabilities because they involve the transfer of both information and care responsibility between clinicians or teams [1,2]. The Joint Commission has also identified inadequate handoff communication as a longstanding patient-safety problem requiring standardized processes and opportunities for questions between the sender and receiver [3].

The ED-to-inpatient transition is especially high risk because it is not only a physical movement from the ED to a ward, high-dependency unit (HDU), or intensive care unit (ICU), but also a transfer of diagnostic reasoning, therapeutic responsibility, pending investigations, and escalation thresholds. Qualitative work on ED-to-inpatient transitions has described failures in information transfer, professional responsibility, and uncertainty management during this boundary [4]. Survey data from US academic emergency medicine programs similarly suggest that ED-to-inpatient handoff practices vary substantially and remain incompletely standardized [5].

The broader emergency medicine and intrahospital handoff literature also supports this concern. An emergency medicine consensus document emphasized that ED handoffs are complex, recurrent patient-safety events that require attention to process design, information content, and outcome measurement [6]. Conceptual work on transitions between emergency physicians and hospital medicine physicians has similarly highlighted the complexity of interunit handoffs across professional cultures and expectations [7]. A systematic review of intrahospital transfer handoffs found consistent evidence that communication breakdown during transfers can affect patient safety and that transfer handoffs have distinct challenges compared with routine shift-to-shift handoffs [8]. Direct observation of ED handoffs has identified communication errors and omissions, while studies of vital-sign communication at ED handoff show that even fundamental severity data may be incompletely transferred [9,10].

Diagnostic error adds a second layer of risk. The National Academies of Sciences, Engineering, and Medicine defines diagnostic error as failure to establish an accurate and timely explanation of the patient's health problem or failure to communicate that explanation to the patient [11]. In the ED-to-inpatient transition, the issue is not only whether the first diagnostic label is correct, but also whether uncertainty, alternative diagnoses, and pending evidence are transferred clearly. Studies of diagnostic error in internal medicine and the cognitive-error literature emphasize that both cognitive and system factors contribute to missed or delayed diagnosis [12-14]. A recent AHRQ systematic review of diagnostic errors in the ED reinforces the importance of diagnostic safety as an emergency care priority, particularly for high-risk presentations and diseases associated with serious harm [15].

This narrative review focuses on ED-to-inpatient transitions in acute medical admissions. It examines common handover failures, diagnostic momentum, medication-reconciliation risks, health-system structural variation, and practical interventions that may improve patient safety.

This review adds to prior ED handoff and transition-of-care literature in three ways. First, it explicitly integrates diagnostic momentum and diagnostic-error concepts with ED-to-inpatient handover failures, rather than treating handover as a communication problem alone [4-7,15]. Second, it frames the ED-to-inpatient transition across health-system structures, including hospitalist-based, acute medical take, consultant-unit, ward, HDU, and ICU pathways. Third, it proposes practical ED-facing frameworks for communicating illness severity, uncertainty, pending tasks, medication risk, and escalation triggers in acute medical admissions.

## Review

Methods

This article is a narrative review. A narrative design was chosen because ED-to-inpatient transitions involve heterogeneous evidence from qualitative studies, observational handoff research, diagnostic-safety literature, implementation studies, professional standards, and health-system descriptions. The review was informed by the Scale for the Assessment of Narrative Review Articles (SANRA), particularly the need for a clear rationale, transparent literature search, appropriate referencing, and evidence-supported argumentation [16].

A non-exhaustive review of recent and seminal English-language literature was conducted between February and April 2026 using PubMed, Google Scholar, AHRQ Patient Safety Network, National Academies resources, and selected professional-body resources. Articles published from January 2000 through April 2026 were considered. Search terms included ED handover, ED-to-inpatient transition, transition of care, acute medical admission, intrahospital transfer, diagnostic momentum, diagnostic error, premature closure, anchoring bias, SBAR (Situation, Background, Assessment, Recommendation), I-PASS (Illness Severity, Patient Summary, Action List, Situation Awareness and Contingency Planning, Synthesis by Receiver), medication reconciliation, hospitalist medicine, acute medical take, and acute medical unit.

Articles and reports were selected when they addressed one or more of the following: ED-to-inpatient handover, intrahospital handoff safety, diagnostic error, diagnostic momentum or cognitive bias, medication reconciliation at admission, structured handover tools, or health-system structures relevant to acute medical admissions. Priority was given to peer-reviewed articles, systematic reviews, major patient-safety reports, and widely cited handover studies. Evidence was weighted according to relevance to ED-to-inpatient transitions, recency, study design, and applicability to acute medical admissions. Where direct ED-to-inpatient evidence was limited, the manuscript explicitly identifies the limitation rather than extrapolating beyond the available data. Because this was not a systematic review, formal risk-of-bias assessment, protocol registration, and quantitative synthesis were not performed.

Results

The ED-to-Inpatient Transition as a Clinical Risk Point

The ED-to-inpatient transition is often viewed operationally: the patient is assigned a bed and moved out of the ED. Clinically, the process is more complex. This review proposes that four elements must move together: the patient, the information, the working diagnosis, and the responsibility for reassessment. This framework synthesizes prior work on ED-to-inpatient failures, ED handoffs, and intrahospital transfer communication, rather than representing a previously validated classification system [4,6-8]. If any one of these elements fails to transfer, the patient may arrive at the ward or ICU without the clinical context required for safe ongoing care.

Unlike elective admissions, acute medical admissions are dynamic. A patient initially labeled as “fever under evaluation” may later declare sepsis, dengue shock, meningitis, pyelonephritis, malaria, or infective endocarditis. A patient admitted as “gastritis” may later prove to have an inferior-wall myocardial infarction. A patient with “dehydration” may actually have sepsis, diabetic ketoacidosis, adrenal crisis, or occult bleeding. Therefore, the ED handover should not merely transfer a diagnostic label; it should transfer the clinical trajectory and the uncertainty attached to that trajectory [4,6,8].

The risk is magnified by the ED workflow. Emergency physicians make decisions under time pressure, interruptions, and crowding, with incomplete data. Laboratory tests, radiology reports, serial troponins, lactate trends, specialist opinions, or cultures may still be pending when the patient leaves the ED. Without a clear ownership plan, pending information can become orphaned.

Common Handover Failures in Acute Medical Admissions

Incomplete transfer of the working diagnosis: A handover that states “admit under medicine for fever” is less useful than one that states: “Fever with hypotension, suspected urosepsis versus dengue; lactate and cultures pending; ceftriaxone and fluids given; reassess blood pressure, urine output, and lactate within one hour.” The receiving team needs to know not only the most likely diagnosis, but also the alternative diagnoses that remain clinically plausible.

This distinction matters because many acute medical admissions begin with nonspecific presentations such as weakness, dizziness, vomiting, abdominal pain, syncope, breathlessness, or altered sensorium. If the ED label is passed on without the reasoning behind it, the inpatient team may inherit a conclusion rather than a differential diagnosis.

Poor communication of illness severity: Vital signs can change rapidly after ED treatment. Patients may appear stable after antipyretics, fluids, oxygen, bronchodilators, insulin, or analgesia. If the receiving team is not told how unstable the patient was before treatment, the risk of deterioration may be underestimated. Studies specifically examining ED handoffs have highlighted omissions in communication of vital signs and other severity markers [9,10].

For example, a patient with sepsis may arrive hypotensive, receive fluids and antibiotics, and reach the ward with a normal blood pressure. Without communication of the initial shock state, fluid requirement, lactate level, urine output, and vasopressor threshold, the inpatient team may not appreciate the need for close monitoring or early escalation.

Pending investigations without clear ownership: Pending investigations are a potentially high-risk component of ED-to-inpatient transfer because they may represent unresolved diagnostic or escalation decisions rather than passive administrative tasks [4,8]. These may include repeat troponin, lactate, renal function, electrolytes, blood gas, blood cultures, urine culture, coagulation profile, computed tomography reports, ultrasound reports, or specialist reviews. The issue is not only that results are pending; it is that responsibility for reviewing and acting on them may be ambiguous.

A safe handover should therefore specify what is pending, why it matters, and who should act. “CT abdomen pending” is weaker than “CT abdomen pending to exclude obstructed infected kidney; urology escalation if hydronephrosis or obstructing calculus is reported.”

Medication reconciliation failures: Medication reconciliation is a formal process for creating the most complete and accurate medication list possible and comparing it with medication orders during care transitions [17]. This is particularly important in the ED because medication histories may be incomplete in elderly patients, unconscious patients, patients with polypharmacy, and patients arriving without records.

Important drug-related risks in acute medical admissions include anticoagulants in bleeding or falls, insulin or sulfonylureas in hypoglycemia, diuretics and renin-angiotensin system inhibitors in AKI, steroids masking infection, and antiplatelets in gastrointestinal or intracranial bleeding. Formal medicine reconciliation within the ED has been associated with reduced medication-error rates in emergency admissions, and studies continue to show important discrepancies between recorded medication lists and patients’ actual use [18,19].

The ED-to-inpatient handover does not need to solve every long-term medication issue. However, it should clearly communicate known high-risk medications, drugs held in the ED, drugs newly started, medications that require urgent review, and medication-related hypotheses for the acute presentation.

Unclear escalation triggers: Escalation plans may be implied rather than stated, particularly when the ED environment is busy and transfer is treated as an operational endpoint. For high-risk medical admissions, the ED handover should include clear triggers: recurrent hypotension, increasing oxygen requirement, worsening sensorium, poor urine output, rising lactate, persistent acidosis, refractory hyperkalemia, recurrent chest pain, or need for ICU review.

Without these triggers, deterioration may be recognized late, especially after transfer to a busy ward. A concise contingency plan changes the handover from a passive transfer of information to an active safety intervention.

Table 1 summarizes common ED-to-inpatient handover vulnerabilities and safer communication elements.

**Table 1:** Common ED-to-Inpatient Handover Vulnerabilities and Safer Communication Elements Source: Created by the author. Author-proposed framework synthesizing literature on ED-to-inpatient handover vulnerabilities and patient-safety communication. ED: emergency department; HDU: high-dependency unit; ICU: intensive care unit

Vulnerability	Unsafe handover pattern	Safer handover element
Working diagnosis	Single label without differential	Most likely diagnosis plus key alternatives and degree of uncertainty
Illness severity	Current stable vitals only	Initial and current vitals, response to treatment, severity category
Pending tests	Results not mentioned or ownership unclear	Pending result, clinical relevance, action owner, escalation threshold
Medication history	Medication list deferred entirely to the ward	High-risk drugs, held drugs, new drugs, suspected drug-related issues
Escalation	"Observe" without triggers	Specific triggers for senior review, ICU/HDU review, repeat labs, or imaging
Receiver understanding	One-way information transfer	Receiver synthesis/read-back of critical plan

Diagnostic Momentum at the ED-to-Inpatient Boundary

Diagnostic momentum occurs when a diagnostic label becomes progressively accepted as it moves through the healthcare system, even when the underlying evidence remains incomplete. It is closely related to anchoring bias and premature closure. Anchoring occurs when clinicians rely too heavily on an initial impression, while premature closure occurs when the diagnostic process stops too early [13,14].

ED diagnostic labels are often provisional by necessity. Emergency physicians must act quickly and frequently start treatment before all data are available. The problem is not the use of working diagnoses; the problem is the failure to communicate that the diagnosis is provisional. Once a patient is admitted under a diagnostic label, subsequent teams may unconsciously treat that label as established fact rather than as a hypothesis to be tested.

This risk is particularly high in common internal medicine presentations. “UTI in elderly patient” can obscure sepsis, stroke, drug toxicity, or metabolic encephalopathy. “Gastritis” can obscure acute coronary syndrome. “Anxiety” can obscure pulmonary embolism or arrhythmia. “Known seizure disorder” can obscure hypoglycemia, hyponatremia, or intracranial bleeding. Diagnostic error literature suggests that both cognitive and system factors contribute to such failures, making handover design a diagnostic-safety intervention rather than a communication exercise alone [11-15].

Table 2 illustrates examples of diagnostic momentum in acute medical admissions with suggested safer handover wording.

**Table 2:** Examples of Diagnostic Momentum in Acute Medical Admissions Source: Created by the author. Author-proposed clinical examples illustrating diagnostic momentum and safer uncertainty communication. ED: emergency department; ECG: electrocardiogram; UTI: urinary tract infection; DKA: diabetic ketoacidosis

Provisional ED label	Potential missed diagnosis	Safer handover wording
Gastritis	Acute coronary syndrome, inferior myocardial infarction	Epigastric pain treated symptomatically; ECG/troponin reviewed; repeat troponin required if pain recurs or risk profile high
Anxiety	Pulmonary embolism, arrhythmia, thyrotoxicosis	Panic-like symptoms but tachycardia persists; PE/arrhythmia not fully excluded
UTI in elderly patient	Sepsis, stroke, metabolic encephalopathy, drug toxicity	Urinary findings present, but altered sensorium may be multifactorial; reassess if no improvement
Viral fever	Dengue shock, malaria, meningitis, sepsis	Fever likely viral, but warning signs/pending platelets/lactate/cultures require review
Dehydration	DKA, adrenal crisis, occult bleeding, sepsis	Volume depletion treated, but acidosis/ketones/hemoglobin/lactate need follow-up
Known seizure disorder	Hypoglycemia, hyponatremia, intracranial bleed, non-convulsive status epilepticus	Seizure history present; metabolic, structural, and ongoing seizure activity still need exclusion

Health-System Structures and Why They Matter

The ED-to-inpatient transition is structured differently across health systems. This affects who receives the handover, how responsibility is assigned, and where ambiguity can occur. The same patient-safety principles apply across systems, but the operational endpoint of the handover differs.

In the United States, many adult non-surgical admissions from the ED are accepted by hospitalist medicine or other admitting services. Wachter and Goldman introduced the modern hospitalist concept in 1996, describing physicians whose work centers on inpatient hospital care [20]. However, the US model is not monolithic: patients may be admitted to hospital medicine, cardiology, neurology, nephrology, gastroenterology, critical care, surgical services, or observation units depending on diagnosis, institutional policy, specialty availability, and bed status. The hospitalist model can provide a defined receiving service for many general medical admissions, but it does not eliminate handover risk because high volume, shift changes, boarding, specialty boundaries, and admission disputes may still fragment responsibility.

In the United Kingdom, acute medical admissions commonly involve an acute medical take or acute internal medicine pathway. Acute Medicine has been described as the “front-door” part of General Internal Medicine, focused on the immediate and early specialist management of adults with urgent or emergency medical problems, typically within the first 72 hours [21]. Acute medical units are designed to provide rapid, definitive assessment and treatment for acute medical emergencies, including patients requiring admission from the ED [22].

In many Indian tertiary-care settings, including consultant-unit models, patients are often admitted under a named consultant, specialty unit, ward, HDU, or ICU, depending on clinical condition, availability, institutional workflow, and family preference. The ED physician may communicate with an on-call consultant, registrar, intensivist, or specialty resident rather than a single hospitalist service. This description should not be read as a uniform national model: workflows differ substantially across corporate hospitals, academic government centers, charitable institutions, specialty hospitals, and tier-2 or resource-variable hospitals. This structure may allow early specialty ownership, but it can also create ambiguity in patients with overlapping problems such as sepsis with AKI, heart failure with renal dysfunction, altered sensorium with metabolic causes, or chest pain with gastrointestinal symptoms. Peer-reviewed handover-safety literature from Indian tertiary care remains limited, and the description offered here is informed primarily by observed institutional practice; this evidence gap is itself an important target for future implementation research in low- and middle-income country emergency systems.

The relevance for a global narrative review is that “ED-to-Internal Medicine” is not universal terminology. “ED-to-inpatient care transition” is more accurate because it includes hospitalist services, acute medical take systems, consultant units, ward teams, HDUs, and ICUs. Studies of conflict between emergency and internal medicine physicians also suggest that disposition, role boundaries, and contextual pressures can shape the handover interaction [23].

Table 3 compares structural variation in ED-to-inpatient transitions across US, UK, and Indian health-system models.

**Table 3:** Structural Variation in ED-to-Inpatient Transitions Source: Created by the author. Author-proposed comparison summarizing structural differences relevant to ED-to-inpatient handover. The comparison is illustrative and does not capture all institutional variation. ED: emergency department; US: United States; UK: United Kingdom; AMU: acute medical unit; HDU: high-dependency unit; ICU: intensive care unit

Setting/model	Typical receiving structure	Potential safety advantage	Potential safety vulnerability
US hospitalist model	Hospitalist/admitting service or specialty service	Defined inpatient generalist ownership	Volume pressure, shift changes, service-boundary disputes
UK acute medical take/AMU	Acute internal medicine team or acute medical unit	Early specialist acute medical assessment	Rapid patient movement and pending-task ownership issues
Indian consultant-unit model	Named consultant, specialty unit, ward, HDU, or ICU	Early specialty involvement and consultant ownership	Ambiguity in multisystem disease and variable handover endpoint
ICU/HDU transfer pathway	Intensivist or critical-care team	Higher monitoring and escalation capacity	Risk if pre-ICU ED course, limits of care, or pending tests are not communicated

Structured Handover Tools

Structured handover tools do not replace clinical judgment. Their value is that they reduce avoidable omissions and create a predictable format for high-risk information. SBAR and I-PASS are among the most widely discussed handover frameworks.

A systematic review found moderate evidence for improved patient safety through SBAR implementation, especially when used to structure communication over the phone [24]. However, the evidence is heterogeneous and should not be interpreted to mean that SBAR automatically improves patient outcomes in every setting. Its effect depends on training, adoption, receiver participation, workflow fit, and whether the tool is used to communicate actionable uncertainty rather than merely complete a form.

In a multicenter study published in the New England Journal of Medicine, implementation of the I-PASS handoff program was associated with reductions in medical errors and preventable adverse events, although the original evidence base was derived primarily from pediatric resident handovers in academic settings [25]. A recent AHRQ rapid review of within-unit intrahospital handoff protocols continues to support structured tools such as I-PASS, while noting that direct evidence specifically for inter-unit ED-to-inpatient transitions remains more limited [26].

I-PASS is particularly relevant to ED-to-inpatient transfers because it explicitly includes illness severity, action lists, contingency planning, and receiver synthesis. ED-specific adaptations of I-PASS have also been explored for ED inter-shift handoffs [27]. Separately, standardized ED admission handoff strategies have been associated with improved verbal handoff quality in patients admitted from the ED [28].

Table 4 applies the SBAR and I-PASS frameworks to an ED-to-inpatient handover, using a clinical example.

**Table 4:** Applying SBAR and I-PASS to ED-to-Inpatient Handover Source: Created by the author. SBAR: Situation, Background, Assessment, Recommendation; I-PASS: Illness Severity, Patient Summary, Action List, Situation Awareness and Contingency Planning, Synthesis by Receiver; ED: emergency department; BP: blood pressure; CKD: chronic kidney disease; AKI: acute kidney injury; ICU: intensive care unit

Framework	Component	ED-to-inpatient example
SBAR	Situation	"65-year-old male with suspected sepsis, currently BP 96/60 after fluids."
SBAR	Background	"Diabetes and CKD; fever and dysuria for three days."
SBAR	Assessment	"Likely urosepsis with AKI; lactate pending; received ceftriaxone and 1.5 L fluids."
SBAR	Recommendation	"Close BP and urine-output monitoring; ICU review if hypotension recurs."
I-PASS	Illness severity	Stable, watcher, or unstable; initial and current vitals.
I-PASS	Patient summary	Presentation, key findings, treatment, response, working diagnosis.
I-PASS	Action list	Pending labs, imaging, repeat troponin/lactate, medication review.
I-PASS	Situation awareness	What could go wrong and what should trigger escalation.
I-PASS	Synthesis by receiver	Receiving clinician repeats back the critical plan, e.g., "So, suspected septic shock, lactate pending; ICU triggers are recurrent hypotension or oliguria; I will repeat lactate at 22:00."

A Practical ED-to-Inpatient Handover Checklist

A safe ED-to-inpatient handover must be brief enough for real ED workflow but complete enough to prevent dangerous omissions. It should not become a long narrative note that no one reads. The goal is to transmit the minimum critical information needed for safe continuation of care.

A useful checklist should include patient identity, illness severity, working diagnosis, diagnostic uncertainty, treatment given, response to treatment, pending investigations, medication risks, escalation triggers, consults, patient/family communication, and receiver confirmation. The World Health Organization has also emphasized communication during patient hand-overs as a patient-safety solution [29].

Table 5 presents a proposed ED-to-inpatient handover checklist for acute medical admissions.

**Table 5:** Proposed ED-to-Inpatient Handover Checklist for Acute Medical Admissions Source: Created by the author. Author-proposed checklist synthesizing patient-safety, handover, medication-reconciliation, and diagnostic-safety concepts for acute medical admissions. ED: emergency department; HDU: high-dependency unit; ICU: intensive care unit; NIV: non-invasive ventilation; ECG: electrocardiogram; ABG: arterial blood gas; VBG: venous blood gas; ACEi: angiotensin-converting enzyme inhibitor; ARB: angiotensin receptor blocker; SGLT2: sodium-glucose cotransporter-2

Domain	What should be communicated
Patient identity and destination	Name, age, ED location, ward/HDU/ICU destination, accepting team or consultant
Illness severity	Stable/watcher/unstable; initial and current vitals; oxygen/vasopressor/NIV status
Working diagnosis	Most likely diagnosis and why it is suspected
Diagnostic uncertainty	Important diagnoses not yet ruled out
Treatment given	Fluids, antibiotics, insulin, oxygen, NIV, vasopressors, analgesia, anticoagulation
Response to treatment	Objective improvement, no response, or deterioration
Pending tests	Labs, cultures, imaging, serial ECG/troponin, lactate, ABG/VBG, specialist review
Medication issues	Anticoagulants, insulin/sulfonylureas, diuretics, ACEi/ARB, steroids, antiplatelets, SGLT2 inhibitors
Escalation triggers	Hypotension, hypoxia, oliguria, altered sensorium, rising lactate, recurrent pain, worsening acidosis
Family/patient communication	Diagnosis explained, risk explained, consent issues, code status if relevant
Receiver confirmation	Receiving clinician repeats key concerns, pending tasks, and escalation plan

Special Risk Groups

Elderly patients: Elderly patients often present atypically. Sepsis may present as delirium, myocardial infarction as weakness, and surgical abdomen as nonspecific discomfort. Frailty, polypharmacy, renal dysfunction, communication barriers, and baseline cognitive impairment increase risk. For these patients, handover should explicitly communicate baseline function, acute change, medication concerns, and family observations.

Patients with sepsis: Sepsis care is trajectory-based. The handover should include suspected source, antibiotics given, cultures taken, fluids administered, lactate status, urine output, mental status, and escalation threshold. A patient who looks temporarily stable after fluids may still be a "watcher."

Patients with chest pain or troponin elevation: Troponin results may be pending or require serial testing. The handover should clarify whether ACS remains a concern, whether ECG changes were dynamic, whether pain recurred, and whether cardiology review is pending.

Patients with AKI or electrolyte disturbance: AKI, hyperkalemia, hyponatremia, and metabolic acidosis require repeat testing and medication review. Failure to communicate repeat-testing plans can delay recognition of deterioration.

Patients with altered mental status: Altered sensorium has a broad differential. Labels such as alcohol intoxication, postictal state, urinary tract infection (UTI) delirium, or psychiatric presentation should be used cautiously unless dangerous metabolic, infectious, neurologic, toxicologic, and structural causes have been considered. Non-convulsive status epilepticus should also remain a consideration in patients with persistent or unexplained altered mental status after an apparent seizure or without a clear metabolic explanation.

Discussion

The ED-to-inpatient transition should be understood as a clinical intervention. It is the moment when diagnostic reasoning, treatment decisions, patient trajectory, and clinical ownership are handed from one team to another. If communication is incomplete, the patient may lose continuity at exactly the point when reassessment is most important.

The literature supports several recurring themes. First, handovers are vulnerable because they combine high workload, time pressure, variable format, interruptions, and shifting responsibility [2-10]. Second, diagnostic safety depends on communicating uncertainty rather than only communicating a label [11-15]. Third, medication reconciliation is a core safety issue during admission transitions [17-19]. Fourth, structured tools such as SBAR and I-PASS can improve reliability, but only if implemented with training, workflow integration, and receiver participation [24-28].

Health-system structure should shape the handover design. A hospitalist-based US model, a UK acute medical take, and an Indian consultant-unit model do not have identical workflows, and each category contains substantial institutional variation. However, they share the same core risk: a patient may move faster than the information needed to care for that patient. The wording “ED-to-inpatient care transition” is therefore preferable to “ED-to-Internal Medicine handover” in a global review because it includes ward, HDU, ICU, hospitalist, acute medicine, and consultant-unit destinations.

Hospitals should consider brief ED admission templates that prompt documentation of severity, uncertainty, pending results, action list, and contingency planning, while avoiding excessive documentation burden. Institution-wide handoff standardization efforts have been described, but implementation must be locally adapted [30]. Hospitalist handoff recommendations similarly emphasize that standardization, written support, face-to-face or interactive communication, and minimal interruptions can improve safety [31].

Cognitive debiasing strategies may also be relevant. Emergency physicians and inpatient teams should treat provisional diagnoses as hypotheses, especially when the patient's course does not fit the initial label. Strategies such as diagnostic time-outs, explicit differential diagnosis, and reassessment triggers may reduce anchoring and premature closure when embedded into clinical workflow [32]. Emergency medicine standards also emphasize prompt assessment, recognition of serious illness, stabilization, and safe onward care for undifferentiated emergency patients [33].

Limitations

This review has limitations. It is a narrative review and does not include systematic screening, risk-of-bias assessment, or quantitative synthesis. The literature on ED-to-inpatient handover is heterogeneous and often institution-specific. Much of the evidence on structured handover tools comes from academic, pediatric, resident-training, or within-unit settings, which may not fully generalize to all ED-to-inpatient transfers. The cross-system comparison is also structurally unequal because the US and UK sections are supported by more published literature than the Indian consultant-unit discussion.

Health-system comparisons in this article are intended to clarify structural differences rather than provide a comprehensive policy analysis. The Indian consultant-unit model is described as a commonly encountered structure in many tertiary-care settings, but practice varies substantially by hospital, specialty availability, staffing, and local admission policy.

Future directions

Future work should evaluate ED-to-inpatient handover tools in diverse health-system settings, including consultant-unit models, resource-variable hospitals, and institutions with prolonged ED boarding. Studies should measure clinically meaningful outcomes such as delayed escalation, missed critical results, medication discrepancies, unexpected ICU transfer, diagnostic revision, mortality, length of stay, readmission, and short-term adverse events.

Implementation research should also examine which handover elements are essential and which create a documentation burden. A practical ED handover tool must be concise, receiver-friendly, and compatible with local staffing patterns. The safest tool is not necessarily the longest tool; it is the one that reliably transfers uncertainty, pending tasks, and accountability.

## Conclusions

The ED-to-inpatient transition is a critical patient-safety boundary in acute medical admissions because diagnostic reasoning, therapeutic responsibility, pending tasks, and clinical trajectory must transfer with the patient. Failures in this process can contribute to diagnostic momentum, missed results, medication errors, unclear escalation, and preventable deterioration. Emergency physicians should communicate illness severity, working diagnosis, diagnostic uncertainty, treatment given, response to treatment, pending investigations, medication risks, and escalation triggers. Receiving teams should confirm understanding and assume explicit ownership of follow-up tasks. Structured tools such as SBAR and I-PASS can support this process, but they must be adapted to local workflow and health-system structure.

Regardless of whether the receiving pathway is a hospitalist service, acute medical take, consultant unit, ward team, HDU, or ICU, the goal is the same: clinical clarity and accountability should not be lost during transfer. ED-to-inpatient handover should therefore be treated as an active patient-safety intervention, not merely an administrative step.
